# Compensatory mechanism of attention-deficit/hyperactivity disorder recovery in resting state alpha rhythms

**DOI:** 10.3389/fncom.2022.883065

**Published:** 2022-09-07

**Authors:** Chuanliang Han, Tian Wang, Yujie Wu, Hui Li, Encong Wang, Xixi Zhao, Qingjiu Cao, Qiujin Qian, Yufeng Wang, Fei Dou, Jian K. Liu, Li Sun, Dajun Xing

**Affiliations:** ^1^State Key Laboratory of Cognitive Neuroscience and Learning, Beijing Normal University, Beijing, China; ^2^IDG/McGovern Institute for Brain Research, Beijing Normal University, Beijing, China; ^3^College of Life Sciences, Beijing Normal University, Beijing, China; ^4^Peking University Sixth Hospital/Institute of Mental Health, Beijing, China; ^5^National Clinical Research Center for Mental Disorder and Key Laboratory of Mental Health, Ministry of Health, Peking University, Beijing, China; ^6^Beijing Key Laboratory of Genetic Engineering Drugs and Biotechnology, Beijing Normal University, Beijing, China; ^7^School of Computing, University of Leeds, Leeds, United Kingdom

**Keywords:** attention-deficit/Hyperactivity disorder, alpha oscillation, multiple components, resting state, model fitting

## Abstract

Alpha rhythms in the human electroencephalogram (EEG), oscillating at 8-13 Hz, are located in parieto-occipital cortex and are strongest when awake people close their eyes. It has been suggested that alpha rhythms were related to attention-related functions and mental disorders (e.g., Attention-deficit/hyperactivity disorder (ADHD)). However, many studies have shown inconsistent results on the difference in alpha oscillation between ADHD and control groups. Hence it is essential to verify this difference. In this study, a dataset of EEG recording (128 channel EGI) from 87 healthy controls (HC) and 162 ADHD (141 persisters and 21 remitters) adults in a resting state with their eyes closed was used to address this question and a three-gauss model (summation of baseline and alpha components) was conducted to fit the data. To our surprise, the power of alpha components was not a significant difference among the three groups. Instead, the baseline power of remission and HC group in the alpha band is significantly stronger than that of persister groups. Our results suggest that ADHD recovery may have compensatory mechanisms and many abnormalities in EEG may be due to the influence of behavior rather than the difference in brain signals.

## Introduction

Alpha rhythm (8-13 Hz) is prominent in the parieto-occipital electroencephalogram (EEG) of awake humans. Alpha power increases with the eyes closed but attenuates on eye-opening (Berger, 1929; Niedermeyer, 1999; Draguhn and Buzsáki, 2004). Alpha-band activity was modulated by visual attention (Hanslmayr et al., 2011; Klimesch, 2012; Weisz et al., 2014; Snyder et al., 2015; Capotosto et al., 2017) and memory load (Palva and Palva, 2007; Freunberger et al., 2009; Sauseng et al., 2010; Foster et al., 2017). Furthermore, alpha has been considered to provide feedback connections among visual areas in both macaques and humans (Wang, 2010; Van Kerkoerle et al., 2014; Samaha et al., 2015; Mejias et al., 2016; Michalareas et al., 2016; Helfrich et al., 2017). The alpha-band activities were also thought to be related to attention-related mental deficits like attention-deficit/hyperactivity disorder (ADHD) (Ter Huurne et al., 2013; Mazaheri et al., 2014).

Despite over 50% of children with ADHD continuing to show symptoms in adulthood (Lara et al., 2009), adult ADHD is less well understood. Hoping to use brain oscillations in different frequency bands (delta, theta, alpha, and beta bands) as biomarkers for brain disorders, many studies have been looking for oscillatory biomarkers for ADHD adults (Clarke et al., 2008; Giertuga et al., 2017). However, many previous studies showed inconsistent results. Taking alpha band in the resting state as an example, some studies showed reduced alpha power associated with ADHD (Woltering et al., 2012; Li et al., 2019), and some indicated that the ADHD patients showed an increase in alpha power (Koehler et al., 2009), and some others showed that alpha power is not significantly changed in ADHD patients (Clarke et al., 2008). There are two possibilities that might cause this inconsistency, one reason might be the sample size in these previous studies was relatively small, and another one might be the relatively rough measurement of the power in the alpha frequency band since these studies did not dissect narrowband and broadband power in the spectrum.

In this paper, we quantitatively studied the difference in alpha-band activity among the ADHD persister, remitter, and healthy control groups, using a three-gauss model to dissect narrowband (component) and broadband (baseline) power in the alpha-band in individuals. By using power spectrum analysis with fine frequency resolution, we found evidence that indicates the existence of more than two oscillators within the alpha band. We then demonstrated how these alpha components and baseline power contributed to differences between ADHD persisters, remitters, and healthy control adults.

## Materials and methods

All participants in EEG experiments gave informed consent to participate in this study. The experiments were conducted in accordance with the principles embodied in the Declaration of Helsinki and approved by the Ethics Committee of Peking University Institute of Mental Health, and Beijing Normal University Institutional Review Board.

### Participants

A total of 261 adults (143 ADHD persisters, 24 remitters, and 94 controls) participated in the experiment, twelve participants were excluded because they did not follow the instructions. Analyzed data were collected from 162 ADHD (141 persisters and 21 remitters) adults (ages 18-39 years old) diagnosed at Peking University; 87 normal healthy controls recruited through campus advertisements also participated in the study (Table 1). ADHD participants fulfilled a diagnosis of adult ADHD through Conners’ Adult ADHD Diagnostic Interview based on the Diagnostic and Statistical Manual of Mental Disorders. All ADHD participants were medication naive. Another current psychopathology was assessed with the Structured Clinical Interview for DSM-IV Axis I Disorders (SCID). Control participants were recruited from local universities and communities, and interviewed to ensure an absence of past or current ADHD. The ADHD Rating Scale (ADHD-RS), Conners’ Adult ADHD Rating Scale–Self-Report Screening Version, and SCID were applied for assessing all participants. All the control participants had no current or previous psychiatric disorders. All participants were of Chinese Han descent.

**TABLE 1:** Subject information.

Items	Persistent ADHD, *n* = 141	Remitters, *n* = 21	Healthy control, *n* = 87	F/χ^2^	*P*
Sex Ratio	MZF:1,71	MZF:6.00	M/F:2.00	4.17	0.124
Age	25.41 (6.00)	18.61 (0.87)	23.96 (4.27)	16.07	<0.001
IQ	118.34 (12.31)	111.29 (11.44)	120.23 (9.91)	5.16	0.006
ADHD symptoms Inattentive	26.65 (3.64)	17.50 (2.28)	13.45 (4.59)	317.00	<0.001
Hyperactivity-impulsive	19.25 (5.06)	15.69 (2.27)	12.13 (4.28)	64.19	<0.001
Total	45.90 (6.70)	33.19 (3.74)	25.57 (8.38)	221.54	<0.001

The table illustrates the basic information of the three groups (note: M is for male, and F is for female).

### Electroencephalogram recordings in the resting state

Participants were seated in a comfortable chair in a dimly lit, electrically shielded room with a low level of environmental noise. Scalp EEG data were recorded continuously with a 128-channel EEG net (Electrical Geodesic Inc., EGI). After they became familiar with the environment, participants were told to close their eyes and stay relaxed and still for 6-10 min (average of 8.84 min), and to do nothing else. Scalp EEG data were recorded at a sampling rate of 1,000 Hz. All electrode impedances were kept under 50 kΩ. Data were referenced to electrode CZ originally and then referenced to a frontal channel near FZ. The stereotypical artifacts, such as eye blinks, eye movements, and muscle tension, were separately removed using the artifact rejection method based on the blind source separation algorithm, Independent Component Analysis (ICA) (Delorme and Makeig, 2004; Li et al., 2019; Zhao et al., 2020). On average, there are four ICs were selected to remove.

### Data analysis

Data processing was performed in MATLAB^1^ with custom scripts. The original continuous data were high-pass filtered at 0.5 Hz and low-pass at 40 Hz. Both the high-pass and low-pass filters were zero-phased FIR filters (third order Butterworth filter) which filter the data both forward and backward to ensure phase delays introduced by each filter are nullified. The dickey-Fuller test was used to test the stationary property of the data. Power spectra of the EEG signals were calculated using multi-taper methods with 5 tapers. Each epoch lasted 10 s, enabling a precise spectrum with a resolution of 0.1 Hz.

### Three gaussian model

**FIGURE 1:**
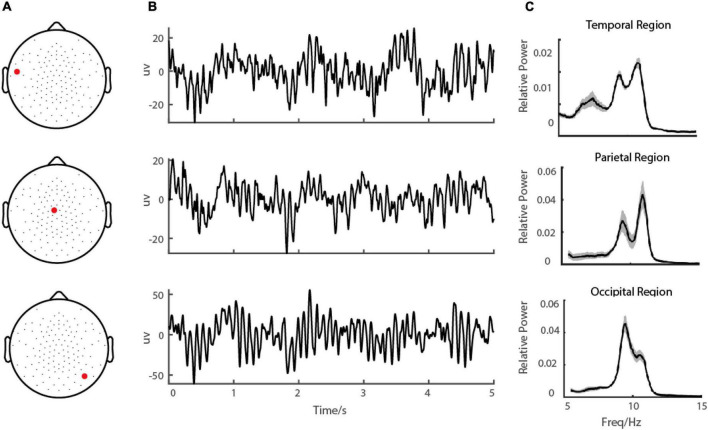
Demo of EEG signal during eye-closed state. **(A)** shows the demo of the channel locations of the EEG, **(B)** shows the EEG signal in a demo electrode in the occipital region and **(C)** shows the corresponding power spectrums.

**FIGURE 2:**
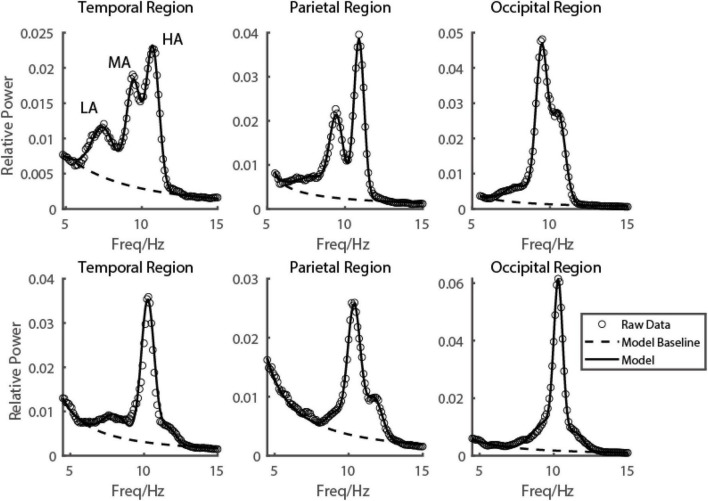
Emergence of multiple alpha rhythms could be well explained by a three-gaussian model. Two subjects’ EEG power spectrums from three example electrodes in three brain regions were shown in the first and second rows, respectively. The raw data was shown in black dots, the fitted curve was shown in a black curve, and the baseline fitted in the model was shown in a black dashed curve.

The Three Gaussian Model is the summation of a baseline and three gaussian functions which represent three component sources in the alpha band for all 128 channels (Figures 1, 2). It is described as follows:


(1) $$Power\left(h,f\right)=Baseline\left(h,f\right)+W_{h,1}\times S\left(1,h,f\right)$$

$$+W_{h,2}\times S\left(2,h,f\right)$$

$$\;+W_{h,3}\times S\left(3,h,f\right)\cdots \cdots$$

(2) $$Baseline\left(h,f\right)=\frac{1}{a_{h}^{f}+b_{h}}+c_{h}\cdots \cdots$$

(3) S(i,h,f)=exp(-(f-μhi)2σhi2)⋯⋯

where S(i,h,f) is the i-th source function in h-th electrode of the signal depending on the frequency f, *W_i_* is the weight of each source, μ*^i^* and σ*^i^* are peak frequency and bandwidth of the i-th source. To evaluate the fitting performance, we calculated the fitting index as follows, the value of which indicated the percentage variance that can be explained by the model. This method has been used in describing gamma-band activity (Han et al., 2020, 2021a,b,c; Wang et al., 2021).


$$FittingIndex=1-\frac{\Sigma {\left(FittingData-RawData\right)}^{2}}{N*\Sigma \left(var\left(FittingData\right)+var\left(RawData\right)\right)}\cdots \cdots \left(4\right)$$

### Statistical analysis

We used the Jarque-Bera test for the normality of the data. The non-parametric ANOVA test (Kruskal-Walis H test) was employed first to test whether the difference exists for the power (baseline and components) among three groups (ADHD persister, remitter, and control), and then the Mann–Whitney U test with Bonferroni correction was used as the post-hoc to check the difference between pairs of groups.

## Results

To characterize alpha-band oscillations in the resting state, we recorded the Scalp EEG from ADHD persister, remitter, and healthy control adults. Participants were told to close their eyes and stay relaxed without doing anything for 6-10 min (Figure 1). For each EEG channel, the recorded data was divided into small segments 10 s in length, and then the power spectrum was estimated by a multi-taper method with a frequency resolution of 0.1 Hz (see Materials and methods).

### Multiple oscillatory peaks found in alpha band

To see the EEG power in the alpha range more clearly on each electrode, with fine frequency resolution (0.1 Hz), two or even three frequency peaks were visible on many electrodes from temporal (Figure 2 first column), parietal (Figure 2 second column) to occipital (Figure 2 third column) lobe. Based on carefully scrutinizing our data (*N* = 249), we found that it was typical that there were three frequency components in the narrow range of the alpha band (low alpha (LA), medium alpha (MA), and high alpha (HA)). Their range was defined based on the value orders of their peak frequencies fitted by the descriptive model. Low alpha was defined as the alpha with the lowest peak frequency (LA: mean = 8.42 ± 0.94 Hz), and the high alpha was defined as the alpha with the highest peak frequency (HA: mean = 11.81 ± 0.84 Hz); the medium alpha was defined as the one between LA and HA (MA: mean = 10.15 ± 0.76 Hz).

### Dissecting different components in the alpha band

**FIGURE 3:**
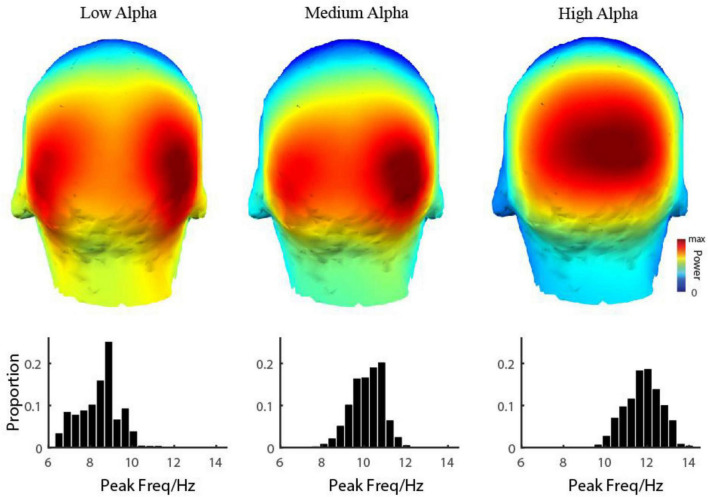
3D Topographic map of three alpha components. The **first row** shows the spatial distribution of the power of three alpha components was shown, respectively. The **second row** shows the distribution of the peak frequency of three alpha components.

Based on the observation in our database, we hypothesized that for each individual subject, the EEG power in the resting state in the alpha range on all 128 electrodes could be modeled by the sum of three frequency components. In more detail, the EEG power in the alpha range was a weighted sum of three frequency components and a baseline (Figure 1 dashed line). The frequency profiles of the three components were all modeled as Gaussian functions, and the frequency profile of the baseline was modeled as a function decreasing monotonically in frequency (see more details in the Materials and methods section for model and model fitting). The three oscillatory components were LA, MA, and HA, with different peaking frequencies (LA: mean = 8.42 ± 0.94 Hz; MA: mean = 10.15 ± 0.76 Hz; HA: mean = 11.81 ± 0.84 Hz) (Figure 3). We found that the EEG power could be reconstructed very well by this model. Across the whole dataset, the model explained 99.29% variance of the normalized data (99.27, 99.31, and 99.35% in groups of ADHD persister, control, and ADHD remitter, respectively).

### Difference of alpha components and baseline power among attention-deficit/hyperactivity disorder persisters, remitters, and healthy controls

**FIGURE 4:**
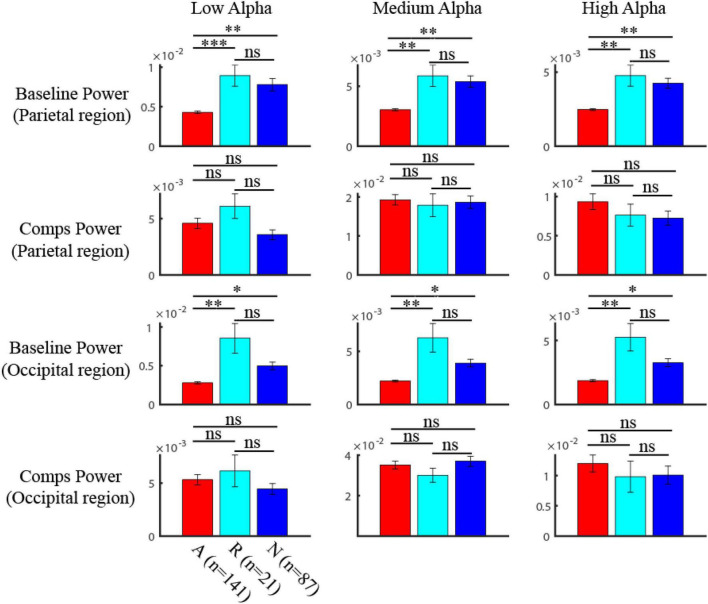
Comparison of the components and baseline power in alpha band among three groups. The baseline power of three groups showed strong significance among three groups (One-way ANOVA) (in parietal region, LA: *p* < 0.001, MA: *p* < 0.001, HA: *p* < 0.001; in occipital region, LA: *p* < 0.001, MA: *p* < 0.001, HA: *p* < 0.001). The multiple comparison of the baseline power in three alpha bands after one-way ANOVA test was shown in the first (parietal region) and third (occipital region) row, where red is for ADHD group, blue is for HC group, and light blue is for remission group (ns: no significance, * *p* < 0.05, ^**^ *p* < 0.01, ^***^ *p* < 0.001). The power of alpha components was not significantly (One-way ANOVA) different among three groups (in parietal region (second row), LA: *p* = 0.11, MA: *p* = 0.98, HA: *p* = 0.18; in occipital region (fourth row), LA: *p* = 0.18, MA: *p* = 0.62, HA: *p* = 0.51).

We further asked whether the powers of alpha components and baseline showed significant differences among ADHD persister, remitter, and normal group by the non-parametric one-way ANOVA test (Kruskal-Walis H test). We found that the power of alpha components was not significantly different among the three groups by one-way ANOVA test (Figure 4; in parietal region, LA: *p* = 0.11, MA: *p* = 0.98, HA: *p* = 0.18; in occipital region, LA: *p* = 0.18, MA: *p* = 0.62, HA: *p* = 0.51). Instead, the baseline power of the three groups showed strong significance among three groups by one-way ANOVA test (Figure 4; in parietal region, LA: *p* < 0.001, MA: *p* < 0.001, HA: *p* < 0.001; in occipital region, LA: *p* < 0.001, MA: *p* < 0.001, HA: *p* < 0.001). In specific multiple comparison, the ADHD persister group is significant smaller than that of remitter (Figure 4; in parietal region, LA: *p* = 0.0028, MA: *p* = 0.0034, HA: *p* = 0.0017; in occipital region, LA: *p* = 0.047, MA: *p* = 0.040, HA: *p* = 0.029) and health control group (Figure 4; in parietal region, LA: *p* < 0.001, MA: *p* = 0.0066, HA: *p* = 0.0074; in occipital region, LA: *p* = 0.0011, MA: *p* = 0.0024, HA: *p* = 0.0014).

## Discussion

With the increasing number of studies on alpha rhythm and its relationship to cognitive functions, it is important to understand the basic properties of alpha. In this study, we developed a model to dissect three oscillators in the alpha range (8-13 Hz) in individual humans. The model enabled us to characterize the properties of distinct alpha components among different groups of human subjects. Our results showed that no significant difference in alpha was found. However, the baseline of the spectrum is significantly different. Our results not only suggest a compensatory mechanism for ADHD recovery in brain oscillations but also demonstrate that dissecting distinct narrow-band oscillatory components is a necessary step for understanding their relation to cognitive functions and brain disorders.

### Compensatory mechanism of attention-deficit/hyperactivity disorder recovery

There are some possible perspectives on the mechanisms of ADHD remission (Sudre et al., 2018). Some researchers found evidence of a convergence mechanism (Schneider et al., 2010; Shaw et al., 2013; Hoogman et al., 2017; Schulz et al., 2017) that views AD/HD as a neurodevelopment defect and the rectification of early anomalies in brain structure with age contributes to the relief of clinical symptoms (El-Sayed et al., 2003). Others proposed that the neural anomalies of AD/HD leave an indelible mark on the brain that will persist across the lifespan regardless of the clinical effect of AD/HD, and remitters recruit new brain systems that allow effective compensation for AD/HD symptoms (Proal et al., 2011; Francx et al., 2015) which is referred to as the “fixed trait” and compensation mechanism. Our results found that the power of alpha components was not significantly different among the three groups. Instead, the baseline power of the remission group in the high alpha band is significantly smaller than that of persisters and healthy controls. This suggests that ADHD recovery may have a compensatory mechanism.

Our work detected a decreased baseline power in the high alpha band in the ADHD remission group. The previous inconsistent results might be due to a blurring of multiple oscillatory components in the alpha band. We should also notice that for the amplitude of alpha components, there is no significant difference among ADHD persisters, remitters, and normal adults, which may indicate that these alpha components are modulated by some other tasks. Further, dissecting oscillatory components not only increases the sensitivity of specific functions related to alpha but also creates a more precise frequency target for neurofeedback, which has attracted more and more attention recently for the treatment of brain disorders including ADHD.

### Multiple distinct alpha oscillation-band vs. sub-bands of alpha

With the increasing number of studies on alpha oscillation, it has been suggested that there existed two or even three sub-bands of alpha oscillation (Klimesch, 1999; Makeig et al., 2002), which might be related to different cognitive functions. Previous results have shown that the power in these different sub-bands (upper and lower alpha) also differed in tasks requiring visual attention (Ding et al., 2006; Sadaghiani et al., 2010; Liu et al., 2016) and memory (Gruber et al., 2005; Michels et al., 2008; Hanslmayr et al., 2012; Elmer et al., 2015; Barnes et al., 2016), and also in the brain’s network (Murias et al., 2007; Sadaghiani et al., 2012), mental disorders (Stoffers et al., 2007; Poil et al., 2014; Yu et al., 2017), neurofeedback training and resting state (Manshanden et al., 2002; Thorpe et al., 2016). Some animal and human studies also suggested that different cortical regions could generate their own alpha oscillations (Lopes Da Silva et al., 1977; Bollimunta et al., 2008, 2011; Scheering et al., 2016). More specifically, previous works have shown that alpha rhythms could be dissected into two components in scalp EEG (Chiang et al., 2011; Barzegaran et al., 2017; Knyazeva et al., 2018). Our results showed that three components are necessary to reconstruct the power spectrum around the alpha band for most individuals. This suggests that alpha contains at least three distinct and significant oscillatory components in the resting EEG, a result that is consistent with the three sub-bands concept. However, our results also suggest that the way to divide multiple oscillatory components based on fixed frequency bands/ranges with respect to the alpha peak frequency might not be precise; one precise way to divide these components should be based on their oscillatory properties, such as peak frequencies and bandwidths in the power spectrum. The theory of Alpha suppression suggests that many cortical regions can generate alpha rhythm when the main rhythm is inactivated (Palva and Palva, 2007) and electrophysiological studies on animals also showed that multiple cortical regions could generate their own alpha oscillation (Lopes Da Silva et al., 1977; Bollimunta et al., 2008, 2011). Therefore, in theory, we might be able to find multiple alphas in the scalp EEG, but practically, our data suggest that three components are enough to capture alpha at the resting state.

### Potential sources for the multiple alpha components

Besides the alpha rhythm in the parieto-occipital lobe, two other rhythms, the mu rhythm and the sensorimotor rhythm (SMR) were also found oscillating in the alpha band (8-13 Hz). The mu rhythm is found in the sensorimotor, motor, and somatosensory cortex (Arroyo et al., 1993; Jones et al., 2010; Liao et al., 2015; Coll et al., 2017). The sensorimotor rhythm (SMR) appears over the sensorimotor cortex (Reichert et al., 2015). Some studies suggested that spectral or topographic properties of the functionally- identified mu rhythm strongly reflect those of upper alpha (Thorpe et al., 2016). Based on previous work on the brain regions that are sources of the EEG (Plattner et al., 2014), our results indicate that low and high alpha is unlikely to be mu and SMR because they are mostly peaking in the parietal lobe, more posterior to sensorimotor, motor and somatosensory cortex. Besides mu and SMR rhythms, alpha oscillation can also be generated by other cortical regions, including multiple areas in the visual cortex (Lopes Da Silva et al., 1977; Bollimunta et al., 2008, 2011).

## Limitations of the study

In this study, there are some limitations should be mentioned. The subjects were Chinese, whose data was collected in Beijing, China. Future studies would collect the data in a wider spatial range. Another limitation is the small sample size of the ADHD remitter group, since the EEG data of the follow-up group is precious, and not easy to collect. Future work would consider more follow-up subjects.

## Data availability statement

The original contributions presented in this study are included in the article/supplementary material, further inquiries can be directed to the corresponding author/s.

## Ethics statement

The studies involving human participants were reviewed and approved by Peking University Institute of Mental Health. The patients/participants provided their written informed consent to participate in this study.

## Author contributions

DX, CH, YW, and LS designed the research. LS, CH, and DX performed the research. CH, HL, EW, XZ, and DX analyzed the data. All authors wrote the manuscript.

## Footnotes

1
www.mathworks.com
